# Long-Term Stress Disrupts the Structural and Functional Integrity of GABAergic Neuronal Networks in the Medial Prefrontal Cortex of Rats

**DOI:** 10.3389/fncel.2018.00148

**Published:** 2018-06-20

**Authors:** Boldizsár Czéh, Irina Vardya, Zsófia Varga, Fabia Febbraro, Dávid Csabai, Lena-Sophie Martis, Kristoffer Højgaard, Kim Henningsen, Elena V. Bouzinova, Attila Miseta, Kimmo Jensen, Ove Wiborg

**Affiliations:** ^1^Department of Clinical Medicine, Aarhus University, Risskov, Denmark; ^2^Neurobiology of Stress Research Group, János Szentágothai Research Centre & Centre for Neuroscience, Pécs, Hungary; ^3^Department of Laboratory Medicine, University of Pécs, Medical School, Pécs, Hungary; ^4^Synaptic Physiology Laboratory, Department of Biomedicine, Aarhus University, Aarhus, Denmark; ^5^Institute of Neuroscience and Physiology, Sahlgrenska Academy, University of Gothenburg, Gothenburg, Sweden; ^6^Department of Health Science and Technology, Aalborg University, Aalborg, Denmark

**Keywords:** depressive disorder, infralimbic cortex, interneuron, learning, NPY, patch-clamp, resilience, chronic stress

## Abstract

Clinical and experimental data suggest that fronto-cortical GABAergic deficits contribute to the pathophysiology of major depressive disorder (MDD). To further test this hypothesis, we used a well characterized rat model for depression and examined the effect of stress on GABAergic neuron numbers and GABA-mediated synaptic transmission in the medial prefrontal cortex (mPFC) of rats. Adult male Wistar rats were subjected to 9-weeks of chronic mild stress (CMS) and based on their hedonic-anhedonic behavior they were behaviorally phenotyped as being stress-susceptible (anhedonic) or stress-resilient. Post mortem quantitative histopathology was used to examine the effect of stress on parvalbumin (PV)-, calretinin- (CR), calbindin- (CB), cholecystokinin- (CCK), somatostatin-(SST) and neuropeptide Y-positive (NPY+) GABAergic neuron numbers in all cortical subareas of the mPFC (anterior cingulate (Cg1), prelimbic (PrL) and infralimbic (IL) cortexes). *In vitro*, whole-cell patch-clamp recordings from layer II–III pyramidal neurons of the ventral mPFC was used to examine GABAergic neurotransmission. The cognitive performance of the animals was assessed in a hippocampal-prefrontal-cortical circuit dependent learning task. Stress exposure reduced the number of CCK-, CR- and PV-positive GABAergic neurons in the mPFC, most prominently in the IL cortex. Interestingly, in the stress-resilient animals, we found higher number of neuropeptide Y-positive neurons in the entire mPFC. The electrophysiological analysis revealed reduced frequencies of spontaneous and miniature IPSCs in the anhedonic rats and decreased release probability of perisomatic-targeting GABAergic synapses and alterations in GABA_B_ receptor mediated signaling. In turn, pyramidal neurons showed higher excitability. Anhedonic rats were also significantly impaired in the object-place paired-associate learning task. These data demonstrate that long-term stress results in functional and structural deficits of prefrontal GABAergic networks. Our findings support the concept that fronto-limbic GABAergic dysfunctions may contribute to emotional and cognitive symptoms of MDD.

## Introduction

Major depressive disorder (MDD) is a common and devastating psychiatric disorder with complex, multifactorial and heterogeneous pathophysiology. Dysfunctional cortical GABAergic networks have been proposed to be causally related to the pathophysiology of MDD (Croarkin et al., 2011; Luscher et al., 2011). This theory was developed based on clinical studies that examined the neocortex of depressed individuals and found reduced GABA concentration using *in vivo* MR spectroscopy (Sanacora et al., 1999; Hasler et al., 2007; Abdallah et al., 2015; Romeo et al., 2018), loss of GABAergic neurons (Rajkowska et al., 2007; Maciag et al., 2010) and indications of deficits in GABA synthesis in post mortem brain tissue (Thompson et al., 2009; Karolewicz et al., 2010).

Our aim was to investigate this GABAergic deficit hypothesis of MDD in a preclinical setting. We used a well-characterized rat model for depression and examined the effect of chronic stress on the morphology and functioning of prefrontal GABAergic neurons. The prefrontal cortex (PFC) is a center for executive and cognitive functions (Miller and Cohen, 2001), and regulates the stress response at several system levels (McKlveen et al., 2013, 2015). Numerous studies have documented that stress has a profound impact on the functioning and plasticity of medial PFC (mPFC) neurons (Holmes and Wellman, 2009; Arnsten, 2015). Pyramidal cells in cortical layers II–III retract their apical dendritic length and reduce their spine density in response to repeated stress exposure (Cook and Wellman, 2004; Radley et al., 2004, 2006b). Also, the GABAergic interneurons undergo dendritic reorganization (Gilabert-Juan et al., 2013) and alter their functioning (Ma et al., 2016; McKlveen et al., 2016; Banasr et al., 2017) in response to stress. Notably, recent optogenetic experiments demonstrated the role of medial PFC circuits in depression-related behavior (Covington et al., 2010; Vialou et al., 2014).

In the present study, we exposed rats to daily mild stressors over a 9-weeks long period and then, we performed functional and morphological analyses to examine the consequences of chronic stress on the structural and functional integrity of GABAergic networks in the mPFC. The cognitive functioning of the animals was assessed using the object-place paired-associate learning task. Six different subgroups of GABAergic interneurons were visualized by immunohistochemistry and systematically counted in all three cortical subareas: anterior cingulate (Cg1), prelimbic (PrL) and infralimbic (IL) cortices. We quantified calretinin-(CR), calbindin-(CB), cholecystokinin-(CCK), parvalbumin-(PV), somatostatin-(SST) and neuropeptide Y-positive (NPY+) neurons. Importantly, these interneuron markers identify distinct subpopulations of GABAergic interneurons, but together they label almost the complete population of GABAergic neurons in the neocortex (DeFelipe, 1997; Kawaguchi and Kondo, 2002; Markram et al., 2004). Potential hemispheric asymmetries were also examined. The functional analysis of GABAergic cells was done by detailed examinations of GABA-mediated synaptic transmission using *in vitro* electrophysiological and pharmacological assays.

Our hypothesis was that chronic stress impairs the structural and functional integrity of prefrontal GABAergic networks. In line with this, we found specific anatomical and electrophysiological evidences for disrupted prefrontal GABAergic networks which associated well with the depressive-like behavior of the animals. At the behavioral level, these deficits were manifested in an impaired performance in a hippocampal-prefrontal-cortical dependent learning task.

## Materials and Methods

### Ethics

All animal procedures were carried out in accordance with the European Communities Council Directive of November 24, 1986 (86/609/EEC) and with university guidelines. The experiments were approved by the Danish National Committee for Ethics in Animal Experimentation (2008/561-447). Throughout the entire experiment adequate measures were taken to minimize pain or discomfort for the experimental animals.

### Animals

Adult male Wistar rats (from Taconic, Denmark) aged 5–6 weeks or 100–120 g upon arrival, were individually housed throughout the experiment, at a 12/12-h light/dark cycle, with food and water *ad libitum*, except when these conditions had to be changed due to the sucrose consumption tests or stress protocol.

Stressed rats were characterized behaviorally with the help of a sucrose consumption test (see below) into two subgroups: depressive-like “anhedonic” and “stress-resilient” animals. For the *in vitro* electrophysiology we used 47 rats (*n* = 16 controls, *n* = 16 anhedonic, *n* = 15 resilient). For the post mortem histology 18 rats were used (*n* = 6 controls, *n* = 6 anhedonic, *n* = 6 resilient). Twenty rats were investigated in the cognitive test (*n* = 10 controls, *n* = 10 anhedonic).

### Behavioral Phenotyping With the Sucrose Consumption Test

**Figure 1:**
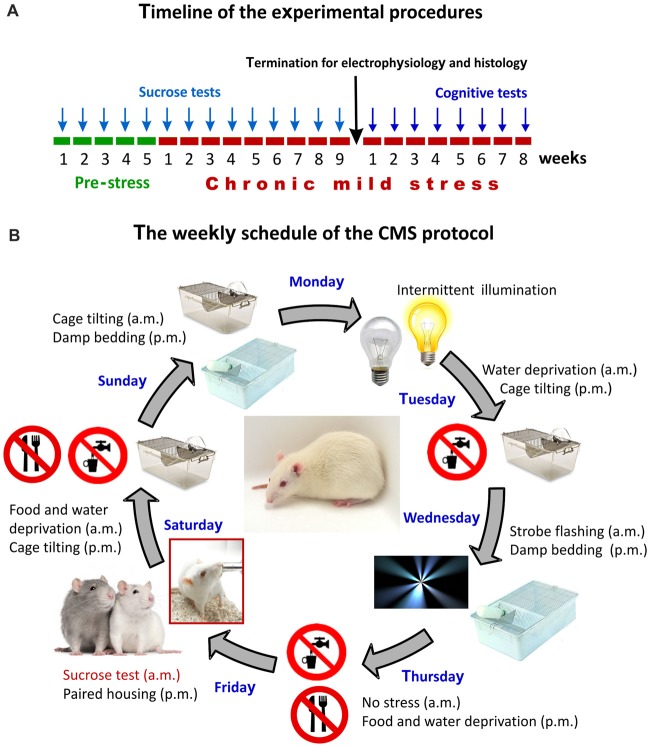
The experimental design and the chronic mild stress (CMS) procedures. **(A)** Timeline of the experimental procedures. Arrows indicate the timing of the behavioral tests. The sucrose test was used for assessing anhedonic-like behavior and the object-place paired-associate learning task was used for evaluating the cognitive performance of the animals. Results of the sucrose consumption test were used to behaviorally phenotype the animals. **(B)** The weekly schedule of the CMS protocol. Every micro-stressor lasted for 10–14 h. Intermittent illumination: light on/off every 2 h; Cage tilting into a 45° position; Strobe flashing: stroboscopic lightning; Damp bedding: pouring water into the cage to damp the beddings; Paired housing: pairing two rats by having an unfamiliar partner at each grouping session. This weekly schedule was repeated every week throughout the 9-weeks of stress exposure.

Animals were trained to consume a palatable sucrose solution (1.5%) for 5 weeks in order to establish their baseline sucrose intake (Figure 1A). Prior to testing, animals were water and food deprived for 14 h. The sucrose consumption tests were performed once a week throughout the entire stress protocol to evaluate the hedonic state of the animals.

### The Chronic Mild Stress (CMS) Protocol

**Figure 2:**
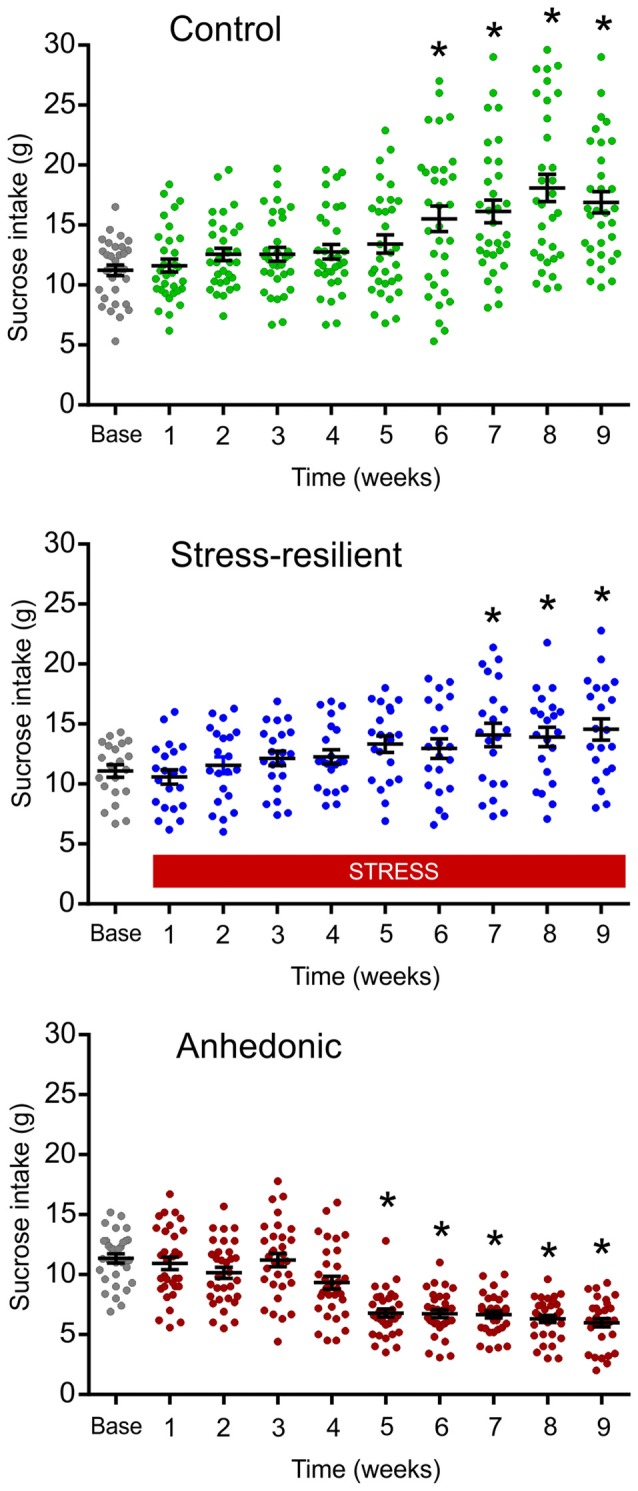
Results of the sucrose consumption test were used to behaviorally phenotype the animals. The graphs display the sucrose consumption of the animals used for the cognitive testing, electrophysiological and histopathological analysis. The repeated testing with the sucrose consumption test enabled us to behaviorally phenotype the animals into stress-resilient and anhedonic rats. Dots represent the individual animals. The anhedonic rats reduced their sucrose intake by more than 30%. A subset of the stressed rats was resistant to the stress and even increased their sucrose intake, similarly to the controls. This group of animals made up the “Stress-resilient” subgroup. Baseline sucrose consumption was defined as the mean sucrose consumption during three sucrose tests conducted before the stress protocol started. There was a significant difference between the groups (*p* < 0.01) and from Week 5 onwards values of the anhedonic group were significantly different from both the control and stress-resilient groups (Tukey’s HSD test: *p* < 0.05). Statistics: repeated one-way ANOVA, followed by Dunnets’s comparison test: *p < 0.05 vs. the base(line) value of the same group.

The CMS is one of the best described and most thoroughly validated animal models for depression (Willner et al., 1992; Willner, 1997, 2005, 2016a,b; Wiborg, 2013). The CMS procedures that were established in our laboratory has been thoroughly described in our previous publications (Henningsen et al., 2009, 2012; Wiborg, 2013; Csabai et al., 2017). Briefly, rats were divided into two matched groups on the basis of their baseline sucrose intake and housed in separate rooms. One group of rats was exposed to 9 weeks of mild stressors. A second group of rats (controls) was left undisturbed. The schedule of the CMS protocol consisted of a fixed sequence of micro-stressors, which were repeated every week. For details of the daily stress schedule see Figure 1B. All the stressors lasted 10–14 h. Based on the results of the sucrose consumption test, the hedonic state of the animals was evaluated and stressed rats were divided into stress-susceptible (anhedonic) rats and stress-resilient rats (Figure 2, for further details on the grouping method see Henningsen et al., 2012). Anhedonic animals are the ones that reduce their sucrose solution intake by more than 30% in response to stress.

### In Vitro Electrophysiology Procedures

Whole-cell patch clamp recordings were obtained from layer II–III pyramidal neurons in the prelimbic-infralimbic region of the mPFC. The neurons were identified in the slices based on their triangular soma with a prominent dendrite projecting to cortical layer I. We investigated spontaneous and miniature inhibitory postsynaptic currents (sIPSCs and mIPSCs), paired-pulse ratio (PPR) of evoked IPSCs, endocannabinoid signaling and GABA_A_ and GABA_B_ receptor mediated currents.

#### Preparation of Brain Slices for Electrophysiology

Animals were anesthetized deeply with isoflurane and then decapitated. The brains were removed quickly from the skull and transferred into ice-cold sucrose solution. Coronal slices (350 μm thick) from the frontal lobe were cut with a Leica VT1200S (Leica Microsystems) vibrating microslicer. Slices containing the prelimbic-infralimbic regions of the mPFC were recovered for 1 h before the recordings in an artificial cerebrospinal fluid (ACSF) media composed of (in mM): 126 NaCl, 2.5 KCl, 2 CaCl_2_, 2 MgCl_2_, 1.25 NaH_2_PO_4_, 26 NaHCO_3_ and 10 D-glucose (osmolality 305–315 mOsmol/kg). To improve slice quality 3 mM kynurenic acid, 0.2 mM ascorbic acid and 0.2 mM pyruvic acid were included in the media during the slicing and storage. All solutions were pH 7.4, when saturated with a mixture of 5% CO_2_–95% O_2_.

#### Electrophysiology

All whole-cell patch-clamp experiments were performed in a recording chamber perfused with ACSF supplemented with 3 mM kynurenic acid (antagonizing inotropic glutamate receptors) at 2–3 ml/min and the temperature was kept at 33 ± 1°C. Neurons were visualized with a help of an infrared-differential interference contrast illumination microscope (Versascope, E. Marton, CA, USA) equipped with a 40× water-immersion objective (Olympus, Ballerup, Denmark, USA) and a CCD100 camera (DAGE-MTI, Michigan City, IN, USA). For *post-hoc* identification, pyramidal neurons were intracellularly labeled in each experiment by 0.5% biocytin included in the pipette solution. Patch pipettes were borosilicate glass types (O.D. = 1.5 mm, I.D. = 0.8 mm; Garner Glass Co., Claremont, CA, USA) using a DMZ-universal puller (Zeitz Instruments GmbH, Munich, Germany). We included only those neurons for further analysis that had a resting membrane potential below −65 mV and the identity of pyramidal neurons was confirmed by examination of firing characteristics in response to injection of depolarizing and hyperpolarizing currents, exhibiting spike-frequency adaptation.

For recordings of GABA_A_R-mediated currents, layer II–III pyramidal neurons were voltage-clamped at a V_hold_ of −70 mV. Patch pipettes had open tip resistances of 4–5 MΩ when filled with intracellular solution containing (in mM): 140 CsCl, 2 MgCl_2_, 0.05 EGTA, 10 HEPES and 0.5% biocytin, adjusted to pH 7.2 with CsOH (280–290 mOsmol/kg). For recordings of miniature IPSCs (mIPSCs) 1 μM of tetrodotoxin (TTX) was added extracellularly.

Tonic GABA_A_R mediated currents were isolated by addition of the GABA_A_ receptor antagonist 2-(3-carboxypropyl)-3-amino-6-methoxyphenyl-pyridazinium bromide (SR95531 > 100 μM) injected into the recording chamber.

For extracellular local stimulation of perisomatic inhibitory fibers, constant current stimuli (0.1–0.2 ms) were applied at 0.05 Hz using a theta-glass electrode, placed approximately 50 μm laterally to the soma of the patched cell. Theta-glass electrodes were filled with ACSF and paired-pulse stimulation (50 ms or 100 ms inter-stimulus intervals) were applied. Stimulation intensity was constant throughout the recording ~20% over threshold.

Postsynaptic GABA_B_R-mediated potassium currents were recorded from layer II–III pyramidal neurons by voltage-clamping the neurons at a V_hold_ of –50 mV. Patch-pipettes were filled with intracellular solution containing (in mM): 130 KOH, 10 KCl, 0.3 EGTA, 10 HEPES, 0.3 Na_3_GTP, 2 MgATP, 5 disodium creatine-phoshate and 0.5% biocytin, adjusted to pH 7.3 with methanesulfonic acid with 285–295 (mOsmol/kg), thereby yielding K-methanesulfonate as the main intracellular constituent. Cell membrane and access resistances were monitored in each experiment with a –10 mV, 50 ms voltage steps. For each experiment neurons were confirmed as regular spiking neurons and resting membrane potentials were noted.

All recordings were carried out using a MultiClamp 700B amplifier (Molecular Devices, Union City, CA, USA). Giga seals (>1 GΩ) were always obtained before break-in in whole-cell recordings. Whole-cell capacitances and access resistances were monitored in each whole-cell experiment. Resistances were compensated by 70%–80% (lag 10 μs), and recordings were discontinued if access resistance changed by more than 20% or exceeded 20 MΩ.

#### Electrophysiological Data Acquisition and Analysis

All recordings were low-pass filtered (8-pole Bessel) at 3 kHz, digitized at 20 kHz, and acquired using a BNC-2110 D/A converter and a PCI-6014 board (National Instruments, Austin, TX, USA) and custom-written LabVIEW 6.1–based software (EVAN v.1.4, courtesy of Istvan Mody, CA, USA), used for analysis of all recordings.

Typical IPSCs detection parameters were a negative deflection from the baseline of at least 5–7 pA for at least 700 μs. All detected events underwent visual inspection and were accepted or rejected before an average of 50–100 events was made, and if obvious events were missed parameters were adjusted to include them. Event amplitude, 10%–90% rise time, frequency and half decay time (T_50%_) were measured, whereas the IPSC weighted decay-time constant (τ_w_) was measured using double-exponential fits and visual inspection of residuals. The PPR was defined as the ratio of the amplitude of the second evoked IPSC (eIPSC) over the amplitude of the first eIPSC.

In order to measure GABA_A_R mediated tonic currents, samples (length 5 ms) were obtained every 100 ms and plotted against time. Contamination of the holding current by spontaneous inhibitory postsynaptic currents (sIPSCs) was removed as described by Nusser and Mody (2002). The mean tonic current was calculated in 4 s-long segments at three time points: just before SR95531 injection (denoted *b*) and 20 s before (*a*) and after (*c*) this time point. The tonic current was taken as *c − b*, whereas the variations in the baseline (*b − a*) were used to assess the stability of the recording. The tonic currents were normalized to the cell capacitance in histograms to report current densities.

#### Solutions and Drugs

Baclofen, CGP55845, kynurenic acid were from Tocris (Avonmouth, UK), while pyruvic acid was from MP Biomedicals (Irvine, CA, USA). All other drugs and reagents were from Sigma (St. Louis, MO, USA).

### Post Mortem Quantitative Histopathology

#### Perfusion, Brain Tissue Preparation and Immunohistochemistry Labeling

After an overdose of sodium pentobarbital (200 mg/ml dissolved in 10% ethanol) animals were transcardially perfused with 0.9% physiological saline followed by 4% paraformaldehyde (pH = 7.4). The brains were removed and postfixed overnight in the same solution at 4°C. Serial coronal sections were cut throughout the entire frontal lobe of the brains using a Vibratome (Leica VT1200S). Sixty micrometer thick sections were collected in series and stored in 0.1 M phosphate buffer (pH = 7.4) with 0.5% sodium azide at 4°C until staining. Immunohistochemical stainings were carried out to label different subgroups of GABAergic interneurons: PV-, CB-, CR-, CCK-, NPY and somatostatin-immunoreactive neurons. Samples from each behavioral group were always processed in parallel to avoid any nonspecific effect of the staining procedure. Six different primary antibodies (Table 1) were used with each we stained one series of every 6th prefrontal sections.

**Table 1:** Primary antibodies used for the immunohistochemistry labeling.

Primary Ab	Dilution	Company
Anti-Calbindin D-28K	1 : 10 000	SWANT Swiss antibodies Cat. Nr.: 300
Anti-Calretinin	1 : 5000	SWANT Swiss antibodies Cat. Nr.: 7699/3H
Anti-Cholecystokinin 8	1 : 5000	AbCam Cambridge UK, Cat. Nr.: ab43842
Anti-Parvalbumin	1 : 10 000	SWANT Swiss antibodies Cat. Nr.: 235
Anti-Neuropeptide Y	1 : 10 000	Peninsula Laboratories International, USA, Cat. Nr.: T-4070
Anti-Somatostatin-14	1 : 10 000	Peninsula Laboratories International, USA, Cat. Nr.: T-4103

Free-floating sections were washed in 0.1 M PBS and then treated with 1% H_2_O_2_ for 20 min. Nonspecific binding was prevented by incubating the sections for 1 h in 5% normal goat serum (Vector Laboratories, Burlingame, CA, USA) in PBS containing 0.5% Triton X-100. Subsequently, the sections were incubated for one night at 4°C with various primary antibodies, for the concentrations see Table 1. After washing, the sections were incubated with a corresponding biotinylated secondary antibody (either biotinylated anti-rabbit or anti-mouse secondary antibody (1:200; Vector Laboratories, Burlingame, CA, USA) for 2 h. Afterwards the sections were thoroughly washed for 1 h, rinsed, incubated in avidin-biotin-horseradish peroxidase (1:200; Vectastaine Elite ABC Kit, Vector Laboratories) for 2 h, rinsed again, and developed for 10 min in diaminobenzidine (1:200; DAB Peroxidase Substrate Kit, Vector Laboratories), and then thoroughly rinsed again. Finally, the sections were mounted on glass slides in a 0.1% gelatin solution and dried overnight, after which they were dehydrated through stepped alcohol washes, cleared in xylene for 30 min and finally coverslipped under DPX (Fluka). Images were acquired on a Nikon Eclipse Ti-U workstation using a 20× and 40× objective.

#### Unbiased Stereological Neuron Quantification

We did an unbiased stereological cell counting to determine the number of different subpopulation of GABAergic neurons in the entire mPFC (Gundersen et al., 1988; West, 1999). Immunolabeled neurons were quantified in the different mPFC sub-areas: anterior cingulate (Cg1), pre-limbic (PrL) and infra-limbic (IL) cortices separately using the MicroBrightField StereoInvestigator (Version 7) cell counting software (Microbrightfield, Colchester, VT, USA). Neuron numbers were estimated with the optical fractionator technique (West et al., 1991; West, 1999). The optical fractionator is an unbiased counting method, which is independent of the size, shape, and orientation of the cells to be counted, and combines the optical disector (Sterio, 1984) with the fractionator-sampling scheme (Gundersen et al., 1988). The parameters of the fractionator-sampling scheme were established in a pilot experiment and were uniformly applied to all animals. The six different subtypes of GABAergic interneurons were counted in six different series of 60 μm thick sections (*f*_1_ = 6) which covered the entire medial prefrontal cortex starting from 4.7 mm to 1.6 mm relative to Bregma, according to the rat brain atlas of Paxinos and Watson (Paxinos and Watson, 2007). On average, 8–9 sections/primary antibody/animal were sampled using a systematic random sampling procedure. Before the quantitative analysis, slides were coded, and the code was not broken until the analysis was completed. Neurons were counted in the three subareas of the medial prefrontal cortex: anterior cingulate (Cg1), pre-limbic (PrL) and infra-limbic (IL) cortices separately. For cell counting we used a Nikon Eclipse Ni-E Motorized Microscope System and the Stereoinvestigator (Version 7) software (Microbrightfield, Colchester, VT, USA). The two hemispheres were investigated separately to evaluate potential hemispheric differences. First, we manually outlined the contours of each cortical subarea using a low magnification objective (×10 lens, NA 0.30). The borders of the cortical areas were identified with the help of a rat brain atlas (Paxinos and Watson, 2007). We also prepared a complete series of Nissl stained sections from the frontal lobe of one animal and this was used to help us to recognize the borders between subareas. Immunolabeled neurons were counted under a ×20 (NA 0.50) objective. The size of the disector frame area, *a*(frame) was 200 μm × 300 μm, and the sampling area, *A*(*x*, *y* step) was 300 μm × 300 μm, yielding: *f*_2_ = 1/(*a*(frame)/1/*A*(*x*, *y* step)) = 1.5. Proper optical dissector rules require guard zones both at the upper and lower surfaces of the section. Here we applied 5–5 μm upper and lower guard zones. After processing the 60 μm thick sections the actual sections thickness was on average 45.12 μm. Thus, the height of the optical disector, *h*, was 45.12–5–*5* = 35.12 μm (*f*_*3*_ = 35.12). After having counted all cells (∑Q^–^) fulfilling the criteria of sampling, the total number of cells was estimated: *N*_total_ = ∑Q × *f*_1_ × *f*_2_ × *f*_3_. Neuron numbers are reported here as total neuron number/hemisphere.

### Cognitive Test: The Object-Place Paired-Associate Learning Task

The different object-place paired-associate learning (dPAL) task was developed as a behavioral touchscreen task in which rodents learn to associate different objects with their specific locations on a screen (Talpos et al., 2009). In each trial, rats were presented with two choices of objects and one blank window simultaneously. After nose poking on the object, being in the correct location, a reward was delivered. Rats were exposed to 75 trials within a 45 min session. As it is shown on Figure 1, the animals were first trained in this learning task for 3 weeks and then, the testing period of 5 weeks started. Daily sessions continued until rats passed a preset criterion of 80% correct touches two days in a row. Among several parameters the maximum number of consecutive correct trials within each session was recorded to have a combined readout on working memory, visual attention and executive functions.

#### Apparatus for the dPAL Task

The Bussey-Saksida touchscreen chamber was from Campden Instruments Limited (Loughborough, UK), and described in detail by Tran et al. (2016). The screen was located in one end of the chamber and a reward tray in the other end. Rewards were 45 mg, dustless precision pellets with bacon flavor from Bio Serv (Flemington, NJ, USA). The screen was covered by a mask with three response windows. Stimulus delivery/detection and operant box inputs/outputs were controlled by ABET II Touch Software (Campden Instruments Ltd., Loughborough, UK).

### Statistical Analysis

Results are presented as the mean ± SEM. Results of the sucrose preference test were analyzed with repeated one-way ANOVA, followed by Dunnets’s Multiple Comparison Test. Electrophysiological data was analyzed using the Student’s independent *t*-test, or one-way ANOVA, followed by Tukey’s Multiple Comparison Test as *post hoc* analysis for further examination of group differences and with the Kolmogorov-Smirnov test. Neuroanatomical data were compared with one-way ANOVA, followed by Tukey’s Multiple Comparison Test as *post hoc* analysis for further examination of group differences. The behavioral data were analyzed applying mixed models for univariate repeated measurements.

## Results

### Long-Term Stress Induced Anhedonic-Like Behavior

Based on the results from the sucrose consumption test, rats were segregated into two sub-groups. One fraction displayed anhedonic-like behavior; whereas the other sub-group consumed the sucrose solution similarly to controls, therefore, this group of animals was named as stress-resilient rats (Figure 2). Animals from these three experimental groups (control, stress-resilient and anhedonic) were used for further electrophysiological, neuroanatomical and behavioral analysis.

### Functional Analysis of GABAergic Synaptic Neurotransmission

To test the hypothesis that long-term stress disrupts GABAergic neurotransmission in the mPFC, we did *ex vivo* whole-cell patch-clamp recordings from layer II–III pyramidal neurons of the prelimbic-infralimbic area. Spontaneous and miniature inhibitory postsynaptic currents, PPRs, endocannabinoid signaling and GABA_A_ and GABA_B_ receptor mediated currents were recorded.

#### Reduced GABAergic Synaptic Input in Anhedonic Rats

**Figure 3:**
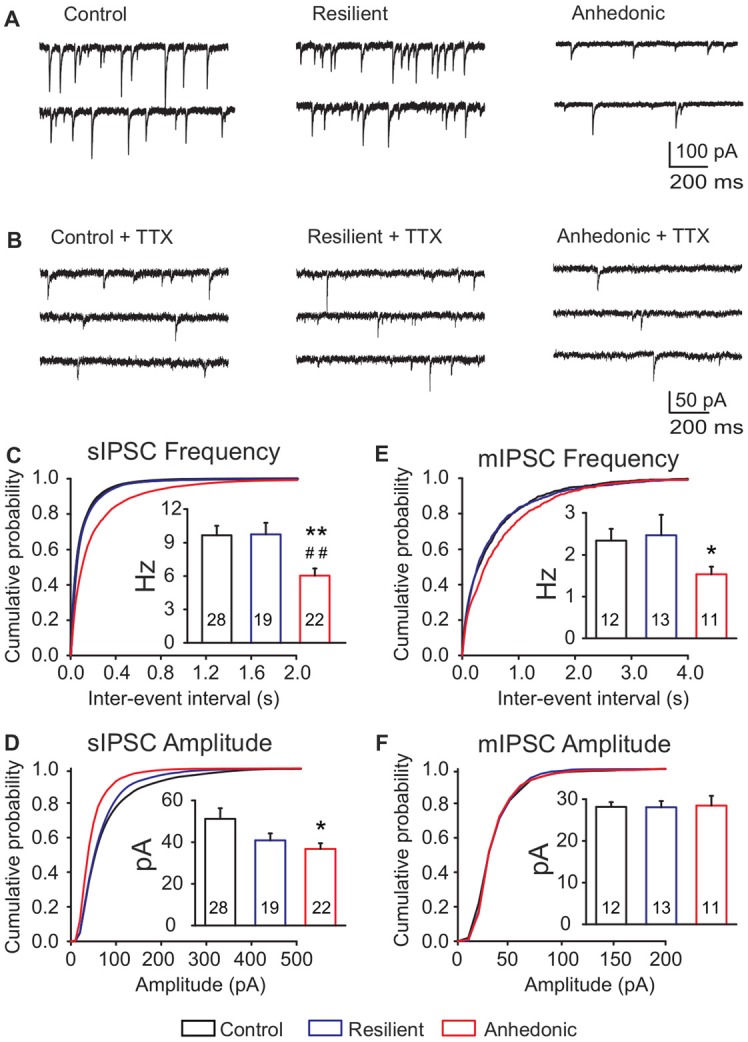
Deficit of GABAergic synaptic neurotransmission in the medial prefrontal cortex (mPFC) of anhedonic rats. Whole-cell patch-clamp recordings of spontaneous inhibitory postsynaptic currents (sIPSCs) **(A)** and miniature inhibitory postsynaptic currents (mIPSCs) **(B)** from layer II–III pyramidal neurons in the ventral mPFC. **(C)** Cumulative probability plot showing altered distribution of sIPSC inter-event intervals in anhedonic rats (Kolmogorov-Smirnov test, *p* < 0.05). Insert in **(C)** Anhedonic rats had reduced mean frequency of sIPSCs. **(D)** Cumulative probability plot displaying altered distribution of sIPSC amplitudes in the anhedonic rats (Kolmogorov-Smirnov test, *p* < 0.05). Insert in **(D)** Anhedonic rats had reduced amplitudes of sIPSCs. **(E)** Cumulative probability plots indicating altered distribution of mIPSC inter-event intervals in anhedonic rats (Kolmogorov-Smirnov test, *p* < 0.05). Insert in **(E)** Anhedonic rats had reduced frequency of mIPSCs. **(F)** Cumulative probability plot showing similar distribution of mIPSC amplitudes in all groups. Insert in **(F)** Mean mIPSC amplitudes were similar between the groups. Data are mean ± SEM. *p < 0.05, **p < 0.01 vs. control group; ^##^*p* < 0.01 vs. the resilient group. The numbers of examined neurons are shown in the columns.

We recorded sIPSCs in the presence of kynurenic acid and found a significant, 37% reduction of sIPSC frequency in anhedonic rats (Figure 3). Average frequencies of sIPSCs were 9.6 ± 0.8 Hz in control (*n* = 28 cells/8 rats), 9.7 ± 1.0 Hz in stress-resilient (*n* = 19 cells/8 rats) and 6.0 ± 0.6 Hz in anhedonic (*n* = 22 cells /9 rats) animals. One-way ANOVA revealed a significant group difference (*F*_(2,66)_ = 6.61, *p* = 0.01) and Tukey’s *post hoc* test yielded significant differences between the anhedonic and control (*q* = 4.61, *p* < 0.01) as well as between the anhedonic and stress-resilient animals (*q* = 4.31, *p* < 0.01). Stress also reduced sIPSC amplitudes. Kolmogorov-Smirnov test revealed a significant difference in amplitude distributions of the anhedonic rats compared to the stress-resilient and control values of sIPSCs (*p* < 0.05). Significant difference was found between the control and anhedonic rats (one-way ANOVA: *F*_(2,66)_ = 3.42, *p* = 0.05, Tukey’s *post hoc* test: *q* = 3.55, *p* < 0.05; Figure 3D). The average amplitude of the sIPSCs was 51.1 ± 5.1 pA in control (*n* = 28 cells/8 rats), 40.8 ± 3.3 pA in stress-resilient (*n* = 19 cells/8 rats) and 36.7 ± 2.7 pA in anhedonic rats (*n* = 22 cells/9 rats).

We also measured mIPSCs in the presence of the Na^+^ channel blocker TTX (1 μM) and found that the frequency was significantly reduced by 35% in anhedonic rats compared to controls (Figures 3B,E,F and Table 2; *t* = 2.18; *p* < 0.05). The average frequency of mIPSCs was 2.3 ± 0.3 Hz in control (*n* = 12 cells/4 rats), 2.5 ± 0.5 Hz in resilient (*n* = 13 cells/3 rats) and 1.5 ± 0.2 Hz in the anhedonic rats (*n* = 11 cells/4 rats). Amplitudes and time parameters of mIPSCs were similar between the groups (Figure 3F and Table 2).

**Table 2:** Amplitude and kinetic parameters of mIPSCs.

	Amplitude, pA	RT_10%-90%_, μs	Decay τ_w_, ms	Frequency, Hz	*n*_cells_	*N*
Control	27.8 ± 1.3	333 ± 6	7.6 ± 0.3	2.3 ± 0.3	12	4
Stress-resilient	27.2 ± 1.6	319 ± 11	6.6 ± 0.5	2.5 ± 0.5	13	3
Anhedonic	28.8 ± 2.5	319 ± 9	7.7 ± 0.6	1.5 ± 0.2*	11	4

Values are mean ± SEM of mIPSC amplitude, rise time, decay kinetics and frequency. Data were obtained from averages of 100–300 consecutive mIPSCs for each cell. Number of neurons (n_*cells*_) and number of rats (N) are shown to the right. *Student’s *t*-test: *t* = 2.18, *p* < 0.05.

#### Decreased Release of GABA From Perisomatic Synapses in the Anhedonic Rats

To assess synaptic release probability at perisomatic synapses, we measured PPRs of evoked IPSCs (eIPSCs) with 50 ms or 100 ms inter-pulse intervals. To activate perisomatic synaptic inputs to the layer II–III pyramidal neurons a theta glass stimulating electrode was placed in the proximal region ~50 μm laterally to the recorded soma.

**Figure 4:**
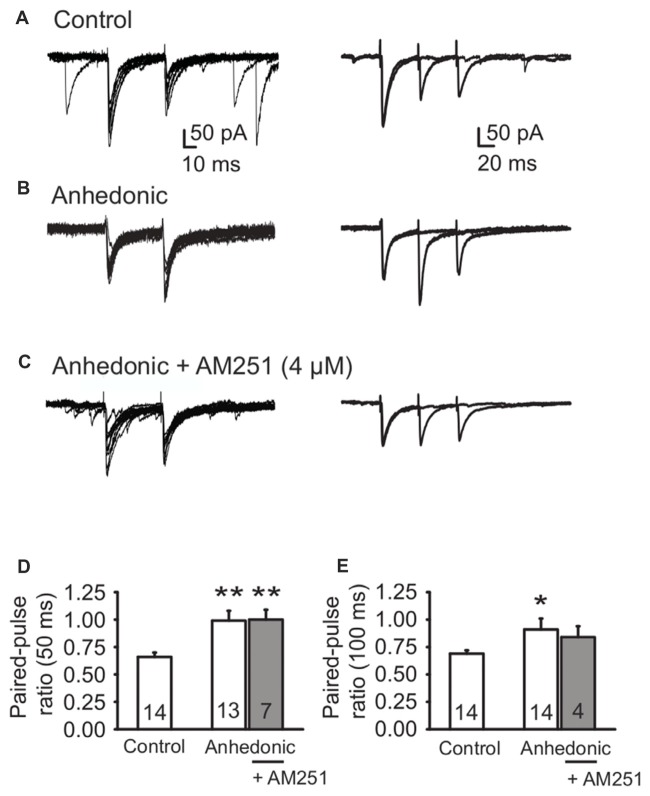
Altered release of GABA from perisomatic synapses in anhedonic rats. **(A)** Representative whole-cell patch-clamp recording of evoked IPSCs (eIPSCs) from a control rat. Paired-pulse depression of eIPSCs (amplitude of eIPSC_2_ relative to eIPSC_1_) was observed. Left panel **(A)** Individual traces of eIPSCs evoked by paired-pulse stimulation with 50 ms interpulse interval. Right panel **(A)** Superimposed average traces of eIPSCs evoked by paired-pulse stimulation with 50 ms interpulse interval and 100 ms interpulse interval, recorded from the same cell. **(B)** Representative recording from an anhedonic rat. The average eIPSC_2_/eIPSC_1_ ratio was increased for both interpulse intervals. Left panel **(B)** individual traces of eIPSCs evoked by paired-pulse stimulation with 50 ms interpulse interval. Right panel **(B)** superimposed average traces of eIPSCs evoked by paired-pulse stimulation with 50 ms interpulse interval and 100 ms interpulse interval, recorded from the same cell. **(C)** Representative recording from an anhedonic rat. The slice was incubated with the inverse agonist of CB1 receptors AM251 (4 μM) for 20 min. AM251 could not rescue the average eIPSC_2_/eIPSC_1_ ratios. Left panel **(C)** individual traces of eIPSCs evoked by paired-pulse stimulation (50 ms interpulse interval) in presence of AM251. Right panel **(C)** superimposed average traces of eIPSCs evoked by paired-pulse stimulation with 50 ms interpulse interval and 100 ms interpulse interval, recorded from the same cell, in presence of AM251. **(D-E)** Histograms showing paired-pulse ratio (PPR) (eIPSC_2_/eIPSC_1_) for interpulse interval of 50 ms **(D)** and 100 ms **(E)**. All data are presented as the mean ± SEM. Statistics: *t*-test *p < 0.05, **p < 0.01 vs. control). The numbers of examined neurons are shown in the columns.

We found significantly increased PPRs in the anhedonic rats at both inter-stimulus intervals (Figure 4). The PPR of eIPSCs at 50 ms interval was 0.66 ± 0.04 (*n* = 14 cells/6 rats) in control and 0.99 ± 0.09 in anhedonic rats (*n* = 13 cells/6 rats; *t* = 3.44; *p* < 0.01). The PPR at 100 ms interval was 0.69 ± 0.03 in controls (*n* = 14 cells/7 rats) and 0.91 ± 0.10 in anhedonic rats (*n* = 14 cells/7 rats; *t* = 2.11; *p* < 0.05).

The endogenous cannabinoid pathway mediates regulation of GABA release via activation of presynaptic CB1 receptors on the axon terminals of CCK+ interneurons (Freund and Katona, 2007). Up-regulation of the endocannabinoid signaling in the mPFC was proposed to be involved in the stress response (Hill et al., 2011) and such change might be responsible for the observed decrease in release probability at the CCK+ neuron terminals in the anhedonic rats. Therefore, we tested the sensitivity of GABAergic neurotransmission to the CB1 receptor inverse agonist AM251. Incubation of the cortical slices from anhedonic rats with AM251 (4 μM) restored the average frequency of sIPSCs to the control level from 5.2 ± 0.83 (*n* = 11 cells/3 rats) to 14.1 ± 3.2 Hz (*n* = 9 cells/3 rats, *t* = 2.95; *p* < 0.01). At the same time, incubation with AM251 (4 μM) did not affect the mean amplitude of sIPSCs, or PPRs of evoked IPSCs in anhedonic rats (Figures 4D,E).

#### Tonic GABA_A_ Receptor Mediated Currents in the Pyramidal Neurons

**Figure 5:**
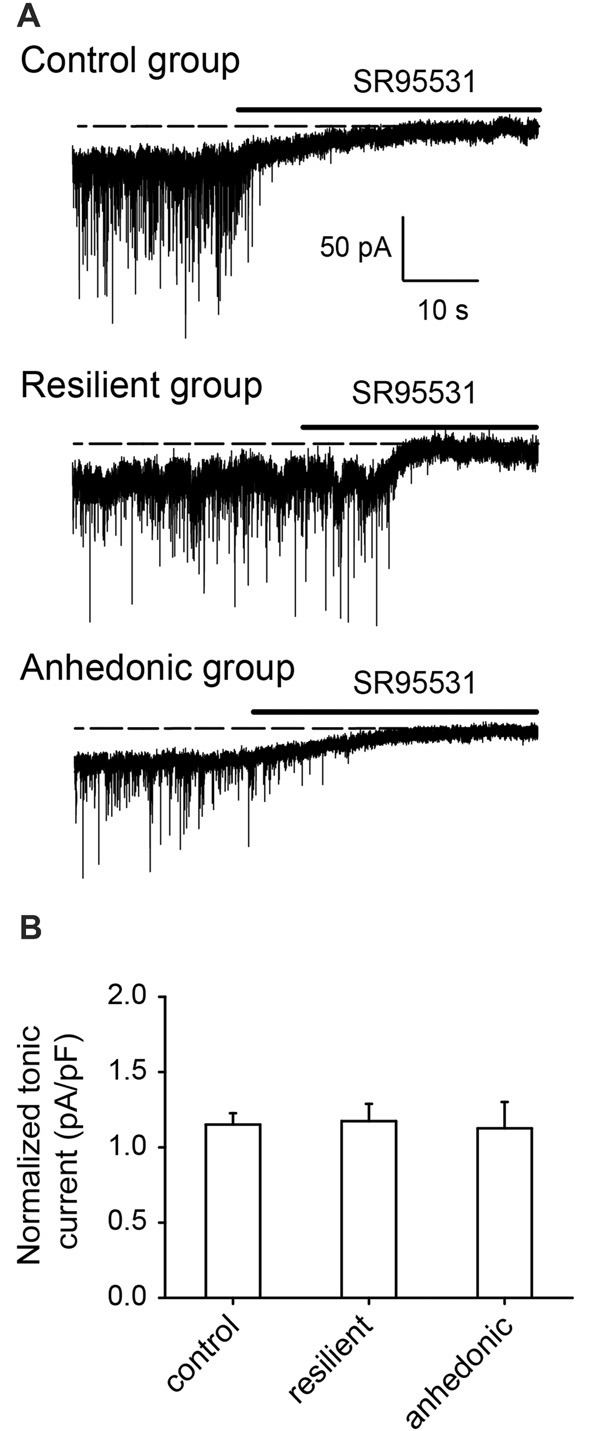
GABA_A_ receptor mediated tonic currents were not affected by stress. **(A)** Whole-cell recordings of GABA_A_ receptor mediated tonic currents. Holding current changes in response to the GABA_A_ antagonist SR95531 were used to estimate the tonic currents in pyramidal neurons of the ventral mPFC. In control rats, the GABA_A_R antagonist induced tonic current was 35.0 ± 2.6 pA (current density = 1.29 ± 0.06 pA/pF, *n* = 9 cells/5 rats), in the stress-resilient rats, tonic current was 27.8 ± 3.2 pA (current density = 1.17 ± 0.11 pA/pF, *n* = 7 cells/6 rats) and in the anhedonic rats 25.1 ± 3.7 pA (current density = 1.05 ± 0.16 pA/pF, *n* = 6 cells/5 rats). **(B)** Histogram showing averages of the GABA_A_ receptor mediated tonic currents normalized on the cell capacitance (current density) from control (*n* = 6 cells/5 rats), resilient (*n* = 7 cells/4 rats) and anhedonic (*n* = 6 cells/4 rats) groups.

To examine whether a decrease in GABAergic neurotransmission affects extrasynaptic GABA_A_R mediated inhibition (Farrant and Nusser, 2005), we measured GABA_A_R mediated tonic currents by recording the outward shift in the holding current after application of the GABA_A_ receptor antagonist SR95531 (>100 μM). GABA_A_R mediated tonic currents were similar in all experimental groups (Figure 5).

#### Reduced GABA_B_ Receptor Mediated Inhibition in the mPFC of Anhedonic Rats

**Figure 6:**
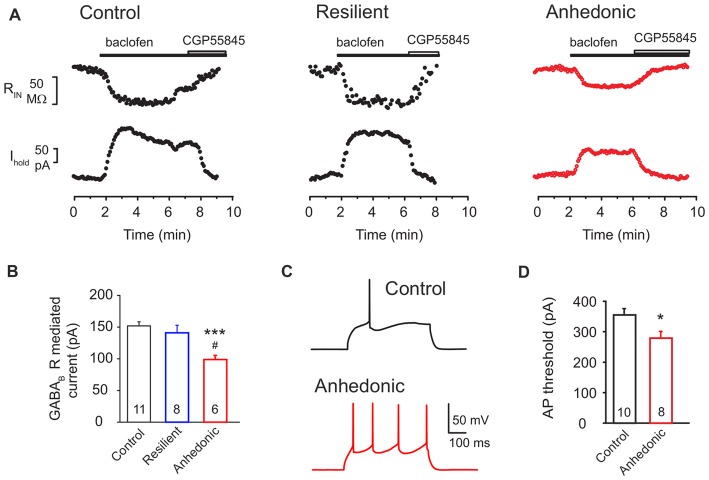
Postsynaptic GABA_B_ receptor-mediated currents are reduced in the anhedonic rats. **(A)** Outward currents evoked by the GABA_B_ receptor agonist baclofen (100 μM) in layer II–III pyramidal neurons (V_hold_ = −50 mV). The baclofen-induced current was associated with a reduced input resistance (R_IN_ upper traces) and was reversed by the GABA_B_ antagonist CGP55845 (8 μM). **(B)** Average GABA_B_R mediated current was significantly reduced in pyramidal neurons of anhedonic rats (*t*-test: ***P < 0.001, vs. control; *t*-test ^#^*P* < 0.05 vs. resilient). **(C)** Spiking elicited by current injection (350 pA/500 ms) in layer II–III pyramidal neurons from control (upper) and anhedonic rats (lower trace). Recordings were done in the presence of the ionotropic glutamate receptor antagonist kynurenic acid (3 mM). **(D)** Average currents required to evoke an action potential (AP) in mPFC pyramidal neurons of control and anhedonic rats (*t*-test, *p < 0.05). The numbers of the recorded neurons are indicated in the columns.

GABAergic inhibition is also mediated by postsynaptic GABA_B_ receptors which are key contributors to the GABAergic signaling in the mPFC (Wang et al., 2010). They provide shunting inhibition in the perisomatic region of the neurons via activation of GIRK channels and inhibition of voltage-dependent calcium channels (VDCCs) (Bettler et al., 2004). Currents in response to the GABA_B_R agonist baclofen (100 μM) were recorded (Figures 6A,B). We found a significant down regulation of the GABA_B_R-GIRK currents in the anhedonic rats compared to controls and to stress-resilient rats. Mean GABA_B_R-GIRK currents were 152 ± 6 pA in control (*n* = 11 cells/6 rats), 141 ± 12 pA in stress-resilient (*n* = 8 cells/6 rats) and 100 ± 5 pA in the anhedonic rats (*n* = 6 cells/5 rats). One-way ANOVA yielded a significant group difference (*F*_(2,22)_ = 9.37, *p* = 0.001) and Tukey’s *post hoc* test found significant differences between the anhedonic and control rats (*q* = 6.01, *p* < 0.001) as well as between the anhedonic and stress-resilient animals (*q* = 4.56, *p* < 0.05; Figures 6A,B). Thus, in addition to a decreased GABAergic synaptic input, we also found down regulation of postsynaptic GABA_B_R signaling in the mPFC of anhedonic rats.

Finally, we found a significantly reduced threshold to evoke an action potential (AP) in anhedonic rats (*t* = 2.56; *p* < 0.05 vs. control, Figures 6C,D), without any significant change in input resistance or single-spike properties (see Supplementary Table S1).

In sum, we found significant evidences of functional GABAergic disturbances in the anhedonic rats which were sensitive to endocannabinoid manipulation. We complemented these data with neuroanatomical investigations and quantified GABAergic cells in the mPFC.

### Stress-Induced Changes in GABAergic Neuron Numbers in the mPFC

**Figure 7:**
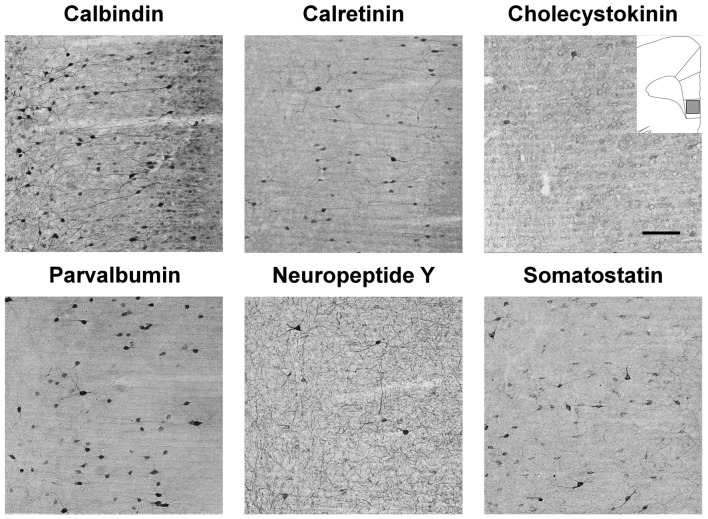
Immunolabled GABAergic interneurons in the infralimbic (IL) cortex of control rats. Representative photomicrographs to demonstrate the results of the immunostaining with the six primary antibodies to identify the different subtypes of cortical GABAergic interneurons. The drawing inserted on the top right with the gray square represents the area where the images were taken. Note that calbindin (CB) is also weakly expressed in the cortical pyramidal neurons, but the GABAergic CB+ interneurons can be identified by their stronger labeling and by their clearly visible dendritic processes which project out in all directions. Scale bar: 100 μm for all images.

**Figure 8:**
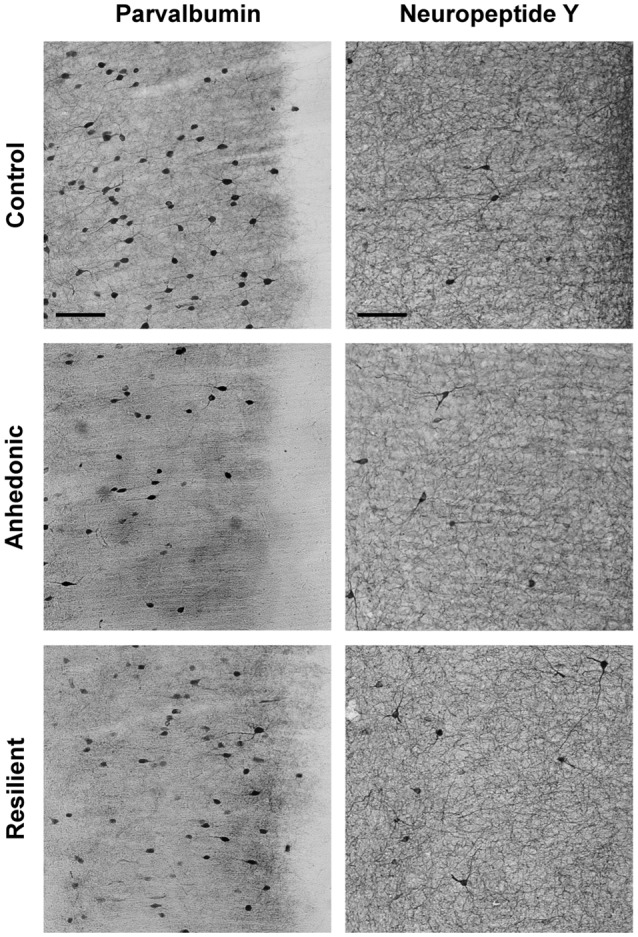
Stress-induced changes in the number of parvalbumin (PV)+ and neuropeptide Y+ (NPY+) neurons in the IL cortex. Representative photomicrographs to demonstrate the differences between the control, anhedonic and stress-resilient animals. The drawing inserted on the top left with the gray square represents the area where the images were taken. Note the lower number of PV+ neurons in the stressed rats, especially in the anhedonic animals. In contrast, NPY+ neurons were more numerous in the stress-resilient rats compared to controls. Scale bar: 100 μm for all images.

**Figure 9:**
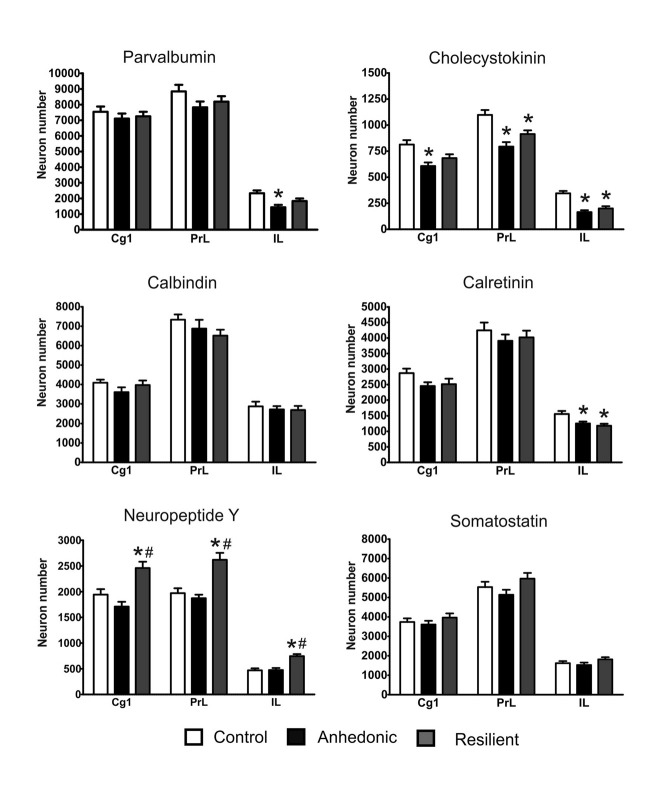
Stress changed the number of GABAergic neurons in the mPFC. Stress had the most pronounced effect on the cholecystokinin (CCK)+ neurons and reduced their numbers in the entire mPFC. The histopathological analysis revealed that the IL cortex was the most susceptible to the stress because in this area stress reduced the number of several types of interneurons, i.e., the PV+, CCK+ and calretinin (CR)+ cells. In most cases, the morphological changes were rather similar in the anhedonic and stress-resilient animals except one cell type, the neuropeptide Y+ neurons. The stress-resilient animals had significantly higher number of NPY+ neurons in their entire mPFC compared to both the control and anhedonic rats. Cell numbers are reported here as total cell numbers in one hemisphere. For individual values see the Supplementary Materials. Statistical analysis: one-way ANOVA followed by Tukey’s multiple comparison test as* post hoc* analysis for further examination of group differences. *P < 0.05 vs. the Control group; # *P* < 0.05 vs. the Anhedonic group. Abbreviations: Cg1: anterior cingulate cortex; IL: infralimbic cortex; PrL: prelimbic cortex.

Representative examples of our immunohistochemistry labelings are shown on Figures 7, 8. We used six different immune-markers to identify cortical interneurons. Together these markers label almost the complete population of GABAergic neurons in the neocortex. Results of the cell quantification data are shown in Figure 9 and the individual data on neuron numbers are listed in Supplementary Tables S2–S7. In general, stress had the most prominent effect on the CCK+ neurons which were reduced in the entire mPFC. The ventral mPFC was the most susceptible part of the mPFC to stress, because in the IL cortex, the numbers of CCK+, CR+ and PV+ neurons were all decreased. Differences between the anhedonic and stress-resilient animals were present only for NPY+ neurons. Stress-resilient rats had higher number of NPY+ neurons in their mPFC than the anhedonic animals.

Stress reduced the number of CCK+ neurons in all three sub-areas of the mPFC of the anhedonic animals and also in the PrL and IL cortices of the stress-resilient rats (Figure 9). In the Cg1, one-way ANOVA yielded a significant group difference (*F*_(2,15)_ = 7.49, *p* < 0.01) and Tukey’s *post hoc* analysis found a significant difference between the control and anhedonic animals (*q* = 5.43, *p* < 0.01). In the PrL cortex, one-way ANOVA found a highly significant group difference (*F*_(2,15)_ = 13.32, *p* < 0.001) and Tukey’s *post hoc* analysis showed significant differences between the control and anhedonic (*q* = 7.25, *p* < 0.001) as well as between the control and stress-resilient rats (*q* = 4.37, *p* < 0.05). In the IL cortex, one-way ANOVA found a highly significant group difference (*F*_(2,15)_ = 20.9, *p* < 0.001) and Tukey’s *post hoc* test revealed significant differences between the anhedonic and control (*q* = 8.63, *p* < 0.001) as well as between the control and stress-resilient animals (*q* = 6.94, *p* < 0.001).

Stress reduced CR+ neuron numbers in the IL cortex of the anhedonic and stress-resilient animals (Figure 9). One-way ANOVA revealed a significant group difference (*F*_(2,15)_ = 6.97, *p* < 0.01) and Tukey’s *post hoc* test found significant differences between the control and anhedonic (*q* = 3.01, *p* < 0.05), as well as between the control and stress-resilient animals (*q* = 4.98, *p* < 0.01).

Stress reduced the number of PV+ neurons in the IL cortex of the anhedonic rats (Figures 8, 9). One-way ANOVA revealed a significant group difference (*F*_(2,15)_ = 7.55, *p* < 0.01) and Tukey’s *post hoc* analysis found a significant difference between the control and anhedonic rats (*q* = 5.48, *p* < 0.01, Figure 9).

We observed significantly higher number of NPY+ neurons in the entire mPFC of the stress-resilient animals (Figures 8, 9). In the Cg1, one-way ANOVA found a highly significant group difference (*F*_(2,15)_ = 12.66, *p* < 0.001) and Tukey’s *post hoc* test revealed significant differences between the control and stress-resilient (*q* = 4.79, *p* < 0.05%), and between the anhedonic and stress-resilient animals (*q* = 6.95, *p* < 0.001). In the PrL cortex, one-way ANOVA found a highly significant group difference (*F*_(2,15)_ = 15.46, *p* < 0.001) and Tukey’s *post hoc* test yielded significant difference between the control and stress-resilient (*q* = 6.28, *p* < 0.01) and between the anhedonic and stress-resilient animals (*q* = 7.24, *p* < 0.001). In the IL cortex, one-way ANOVA found a highly significant group difference (*F*_(2,15)_ = 16.12, *p* < 0.001) and Tukey’s *post hoc* test yielded significant differences between the control and stress-resilient (*q* = 7.03, *p* < 0.001%) and between the anhedonic and stress-resilient rats (*q* = 6.88, *p* < 0.001).

Stress had no statistically significant effect on the number of CB- and somatostatin-positive neurons (Figure 9).

### Cognitive Test: The Different Object-Place Paired-Associate Learning (dPAL) Task

**Figure 10:**
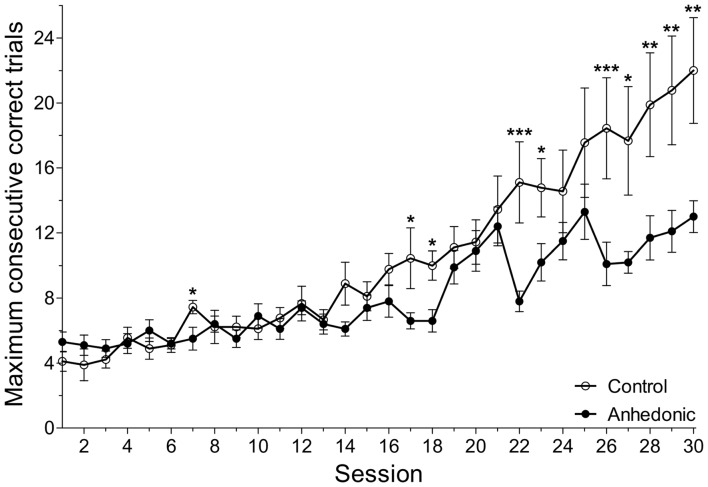
Anhedonic rats were impaired in the object-place paired-associate learning task. The object-place paired-associate learning task is a touchscreen task in which rats learn to associate objects (shapes) based on their specific locations on the touchscreen. Control rats had a gradual improvement in this task and showed a continuous increase in the maximum number of consecutive correct trials. The anhedonic rats were impaired in this task. They could not learn the task, because they could achieve fewer consecutive correct trials. The difference between the two groups was most prominent in the end of the testing period. The number of consecutive correct trials within one session is a combined readout of working memory, visual attention and executive functions. Impaired performance of the anhedonic rats indicates disturbed functioning of hippocampal-prefrontal-cortical neural circuits. Furthermore, we found significant correlation between the sucrose intake of the animals and their performance in this learning task. The x-axis displays the absolute session number. As it is shown on Figure 1, the animals were first trained in this learning task for 3 weeks and then, the testing period of 5 weeks started. During this testing period, every day each animal would perform one session which consisted of maximum 75 task trials or 45 min, whatever would be reached first. Data was log-transformed. Data was compared with repeated measurements mixed model analysis and simple main effects of group were tested for each session. *P < 0.05; **P < 0.01; ***P < 0.001 vs. the Control group in the same Session.

In the dPAL task, the number of consecutive correct trials within one session is a combined readout on working memory, visual attention and executive functions. The maximum number of consecutive correct trials was significantly lower in anhedonic animals compared to control rats (Figure 10). Repeated measurements mixed model analysis revealed a significant interaction effect of group and session with *F*_(29,491)_ = 2.39, *p* = 0.0001. Performance differences between groups were found in session 7, 17, 18, 22, 23, 26–30 (Figure 10). These results show that stress-induced anhedonia associate with cognitive impairments in working memory, visual attention or executive functions. Furthermore, there was a significant correlation between the sucrose intake of the animals and their performance in this learning task. For details see the Supplementary Materials.

## Discussion

The aim of the present study was to examine fronto-cortical GABAergic network functions in a rat model for depression. Exposure to long-term stress produced a depressive-like (anhedonic) behavioral phenotype in a subset of the animals. These rats have pronounced impairments in GABAergic neurotransmission and reduced number of GABAergic neurons in mPFC. In the stress-resilient animals, GABAergic changes were either completely absent or less pronounced, or different compared to the changes detected in the anhedonic rats. In the anhedonic animals, stress reduced the number of CCK-positive perisomatic inhibitory neurons in the entire mPFC and we found reduced numbers of CR- and PV-positive neurons in IL cortex. These data were complemented by the electrophysiological findings which revealed a stress-induced reduction of GABA_A_ receptor mediated synaptic transmission and a synaptic dysfunction resulting in reduced release probability of perisomatic GABAergic synapses in the same brain area. Postsynaptically, we found no compensatory GABAergic upregulations, but an unaffected tonic inhibition and reduced postsynaptic GABA_B_ receptor currents, further compromising inhibitory input onto pyramidal neurons in the anhedonic animals. The post mortem histopathological analysis revealed that stress-resilient rats have higher number of NPY-positive neurons in mPFC compared to controls and anhedonic rats. Finally, we found compromised cognitive capacity of the anhedonic rats in the object-place paired-associate learning task, which indicates an impaired functioning of their hippocampal-prefrontal cortical circuits. Overall, our present data demonstrates that rats with a depressive-like behavioral phenotype have pronounced disturbances both in the morphology and functioning of GABAergic networks in the ventral mPFC.

Numerous clinical data document cortical inhibitory deficits in MDD (e.g., Hasler et al., 2007; Levinson et al., 2010; Croarkin et al., 2011; Luscher et al., 2011; Luscher and Fuchs, 2015). Based on these findings, it has been postulated that fronto-cortical GABAergic deficits contribute to the pathophysiology of depressive disorders. We tested this hypothesis in an animal model which involved long-term stress exposure since chronic-stress models are useful to investigate study the neurobiology of depressive disorders (Willner, 1997; Wiborg, 2013; Czéh et al., 2016).

Diverse subtypes of GABAergic interneurons provide networks of inhibition that sculpt the firing pattern of cortical pyramidal cells and orchestrate their network oscillations that are essential for mediating complex cognitive processes such as perception, learning and memory (Buzsáki and Draguhn, 2004; Somogyi and Klausberger, 2005). A specific subtype of GABAergic neurons that innervate the perisomatic domain of principal neurons has a pivotal role in generating synchronized network oscillations (Klausberger et al., 2005; Somogyi and Klausberger, 2005). PV-positive and CCK-positive neurons are the two major perisomatic inhibitory interneurons and they provide functional dichotomy in the inhibitory network because of their distinct membrane properties, expression patterns of receptors, and their presynaptic modulations (Klausberger et al., 2005; Freund and Katona, 2007). It has been proposed that malfunctioning of the highly modifiable CCK+ interneuron syncytium is likely to result in mood disorders, because these cells carries information from subcortical pathways about the emotional, motivational and general physiological state of the animal (Freund, 2003; Freund and Katona, 2007). Indeed, here we found that CCK+ neuron numbers were reduced in the entire mPFC of the anhedonic animals.

Our observation that stress can decrease the number of immunolabeled neurons has two imaginable explanations: one possibility is that the neurons die because of the stress-induced neurotoxicity, or the intracellular expression level of the specific protein marker is reduced, therefore, it can not be visualized with the immunohistochemistry labeling. We have done TUNEL-staining (data not shown) to detect for apoptosis in the brains of the stressed rats, but we could not find any evidence of cell death. One should note however, that apoptosis is a rapid process. Neurons might have died at an earlier stage, during the 9-week long stress protocol. We have also performed an electron microscopic analysis on brain samples from anhedonic rats, but we did not find any evidence of neuronal death or degeneration (Csabai et al., 2018). Our earlier findings also argue against neuronal cell death. In the same CMS stress model, we found pronounced reduction in the number of PV-positive perisomatic inhibitory neurons (Czéh et al., 2015; Csabai et al., 2017) however, the number of perisomatic inhibitory synapses was not affected by stress (Csabai et al., 2017).

We suggest that the changes in neuron numbers that we observed here are the result of altered expression of the specific cell markers. Stress-induced changes in GABAergic cellular marker expression have been described recently in neurons of the hippocampus and PFC (Banasr et al., 2017). Reduced cytoplasmic protein expression may indicate either a functional impairment or a compensatory molecular response. For example, reduced concentration of cytoplasmic calcium-buffering proteins such as PV and CR most likely reflect a functional impairment of the affected cell. Reduced production of CCK on the other hand may indicate a compensatory mechanism. CCK plays an important role in the stress-response since CCK injection to the mPFC is anxiogenic and can trigger depressive-like behavioral response, whereas the blockade of the CCK-B receptor in the mPFC can induce a stress-resilient phenotype (Vialou et al., 2014).

Our histopathological analysis demonstrated that stress has the most pronounced effect on the IL cortex. In this cortical area, stress reduced the number of PV+, CCK+ and CR+ neurons. Our present data is in line with the well documented concept that the IL cortex is permanently activated during chronic stress (Hinwood et al., 2011; Flak et al., 2012) and that stress results in dendritic remodeling in this sub-area (Izquierdo et al., 2006; Dias-Ferreira et al., 2009; Goldwater et al., 2009). The IL cortex plays an important role in the regulation of an appropriate stress response, emotional control, and depressive-like behavior (Izquierdo et al., 2006; Radley et al., 2006a; McKlveen et al., 2013) and it is well documented that stress disrupts the proper functioning of this cortical area (Izquierdo et al., 2006; Wilber et al., 2011; Koot et al., 2014; Moench et al., 2015; Novick et al., 2015). Stress exposure results in the regression of dendritic material and loss of dendritic spines of layer II–III pyramidal neurons in the IL cortex (Radley et al., 2004; Izquierdo et al., 2006; Perez-Cruz et al., 2007, 2009; Dias-Ferreira et al., 2009; Goldwater et al., 2009; Shansky et al., 2009). Therefore, we made the electrophysiological recordings in this cortical area.

Our earlier studies documented that PV+ and CCK+ neurons of the hippocampus are differentially altered by the prolonged stress (Hu et al., 2010; Czéh et al., 2015; Csabai et al., 2017). Numerous molecular and cellular mechanisms can reduce synaptic vesicle release probability at the synaptic terminals of interneurons, including alterations of the presynaptic VDCCs (Rossignol et al., 2013; Wen et al., 2013), altered positional priming of vesicles (Burnashev and Rozov, 2005; Hefft and Jonas, 2005; Bucurenciu et al., 2008; Han et al., 2011), or deficiency in molecular priming of the GABAergic vesicles (Fernández-Chacón et al., 2001; Maximov and Südhof, 2005; Kerr et al., 2008; Guzman et al., 2010; Bacaj et al., 2013). Recently, we did a gene expression profiling study in laser-captured micro dissected tissue from the granule cell layer of the dentate gyrus from stressed rats and we found positive correlation between the down-regulation of the *Rim1* gene (Rab3 interacting molecule 1) and anhedonic behavioral phenotype (Christensen et al., 2011). Rims and Rim-binding proteins are key organizers of presynaptic active zones (Betz et al., 2001; Schoch et al., 2002) and participate not only in the docking and molecular priming of vesicles (Deng et al., 2011), but also in the tethering of VDCCs to the active zones of glutamatergic and GABAergic synapses (Kaeser et al., 2011). Studies focusing on hippocampal neurons from conditional knockout mice demonstrated that single deletion of *Rim1* or *Rim2* alone can impair the priming of vesicles and GABA release, while double *Rim1*/*Rim2* deletion severely impaired the Ca^2+^ dependance and synchronization of GABA release (Kaeser et al., 2012). In the present study, we found similar GABAergic synaptopathy in the mPFC of the anhedonic animals and it is tempting to suggest, that downregulation of Rim1 was responsible-at least in part-, for the impaired perisomatic GABAergic input. Other molecular mechanisms might also be involved, as our data on proteomic profiling using hippocampal samples from CMS rats demonstrated altered regulation of several proteins associated with the SNARE complex and vesicle recycling (Bisgaard et al., 2007, 2012; Henningsen et al., 2012). In sum, our data suggests that long-term stress alters the efficacy and synchronization of GABA release at the perisomatic synaptic terminals in the mPFC of anhedonic rats.

We also report that long-term stress significantly reduced GABA_B_-GIRK currents of pyramidal neurons in the mPFC of anhedonic rats. Such reduction of GABA_B_-GIRK currents may reflect an altered coupling of G-protein to GIRK channels due to changes in receptor phosphorylation state (Couve et al., 2002; Guetg et al., 2010; Terunuma et al., 2010), or downregulation of RGS (regulators of G protein signaling) proteins (Labouèbe et al., 2007), as well as down regulation of GABA_B_ receptors from the neuronal surface (Guetg et al., 2010; Terunuma et al., 2010; Benke et al., 2012; Padgett et al., 2012). Importantly, several independent studies convincingly demonstrated that the number of membrane GABA_B_Rs can be down regulated in neurons by shift in equilibrium from surface bound to internalized receptors in response to sustained elevation of extracellular glutamate (Guetg et al., 2010; Maier et al., 2010; Terunuma et al., 2010). It is tempting to suggest that this latter molecular mechanism is the explanation-at least in part-, for the plastic down regulation of postsynaptic GABA_B_R signaling in pyramidal neurons in the mPFC of anhedonic rats. The sustained elevation of extracellular concentration of glutamate in the mPFC and hippocampus is likely to contribute to the pathogenesis of MDD and to the cognitive deficits associated with the disorder (Popoli et al., 2011; Musazzi et al., 2012). Notably, in a previous study we reported increased extracellular glutamate levels in anhedonic rats (Delgado y Palacios et al., 2011). In the present study, we did not perform any experiments to specifically test the effects of GABA_A_ or GABA_B_ blockers on excitability. Further studies should investigate the exact molecular mechanisms contributing to the altered GABA_B_R signaling in the anhedonic rats. Finally, it should be noted that GABAergic autapses might be indirectly involved in controlling the excitability of pyramidal neurons (Bacci and Huguenard, 2006), however their involvement in the current setting is still unknown.

The reduced GABAergic electrophysiological responses in the anhedonic rats are in accordance with the structural alterations. The altered interneuron numbers suggest functional impairment of the cells and cause reduced inhibitory input onto principal neurons. In accordance, we found reduced number of inhibitory synapses in the IL cortex of anhedonic rats in a recent quantitative electronmicroscopic study (Csabai et al., 2018). The reduced release probability of perisomatic GABAergic synapses also suggests a synaptic dysfunction, because a significant increase in the PPR in anhedonic rats indicates a reduced release probability of GABA vesicles from perisomatic synapses. Interestingly, this deficit did not lead to compensatory postsynaptic upregulations of inhibition, but rather to the contrary, since the amplitude of GABA_B_ currents were reduced to two-thirds in anhedonic animals. Furthermore, the inability of AM251 to fully rescue the anhedonic rat phenotype points to effects of AM251 on presynaptic interneuron firing, rather than actions on GABAergic nerve terminals, since PPRs were not restored by the endocannabinoid antagonist.

In the present study, the experimental rats were behaviorally phenotyped into stress-susceptible (anhedonic) and stress-resilient groups and we investigated functional and morphological differences at the cellular level that could explain these different behavioral responses. In the electrophysiological experiments there were clear differences between the anhedonic and stress-resilient groups in most cases. In our histopathological analysis, we found significant differences between these two groups only in the number of neuropeptide Y interneurons. The fact that stress-resilient rats have higher number of NPY+ neurons in mPFC also suggests that the neurons apparently change their immune-phenotype during the stress exposure. There have been reports on newborn interneurons in the adult neocortex of rats (Dayer et al., 2005; Cameron and Dayer, 2008), but it is unlikely that so many new NPY+ neurons have been generated during the 9 weeks of stress. Alternatively, stress-resilient rats have a higher number of NPY+ neurons, *a priori*, which may generate a protective effect. There is *in vitro* experimental evidence, that brain-derived neurotrophic factor (BDNF) can specifically stimulate NPY expression in cultured cortical neurons, suggesting that BDNF modulates the NPY expression of GABAergic neurons in the cerebral cortex (Nawa et al., 1993). There is also *in vivo* evidence that chronic infusion of BDNF in the hippocampus can induce a long-lasting increase of NPY expression (measured by immunohistochemistry and radioimmunoassay) whereas a similar BDNF injection did not modify the expression levels of glutamic acid decarboxylase (GAD, the rate-limiting enzyme for GABA synthesis) and somatostatin (Reibel et al., 2000). Parallel to this data, clinical findings demonstrate that the gene expression of NPY is gradually down regulated during early development and there is evidence for altered number of NPY+ neurons in the neocortex of schizophrenic patient (Ikeda et al., 2004; Fung et al., 2010). Overall, these data suggest that the protracted development of specific interneuron subpopulations may be associated with a particular vulnerability to neuropsychiatric disorders (Fung et al., 2010). At this point we speculate that the stress-resilient animals might have acquired more NPY+ neurons in the mPFC during development, which may have a protective effect against stress later in adulthood. Neuropeptide Y is one of the most abundant neuropeptides in the central nervous system and regulates the stress response and emotional behaviors (Morales-Medina et al., 2010; McGuire et al., 2011; Mickey et al., 2011; Kormos and Gaszner, 2013) and has been suggested to be involved in anxiety, depression and posttraumatic stress disorder (PTSD) (Sah et al., 2009; Sah and Geracioti, 2013; Enman et al., 2015). Clinical and pre-clinical studies suggest that NPY has anxiolytic-like and antidepressant-like effects and seems to mediate resilience (Sjöholm et al., 2009; Enman et al., 2015). For example, in an animal model of PTSD it was found that the NPY system is associated with behavioral resilience to stress exposure and suggested that NPY may promote resilience and recovery (Cohen et al., 2012). A recent study demonstrated that central NPY via the Y1 receptor plays an important role in mediating the adaptation mechanism against chronic stress (Yang et al., 2018). Furthermore, NPY+ neurons of the mPFC seem to play a special role in the long-range inhibitory circuitry (Saffari et al., 2016).

Recently, McKlveen et al. (2016) reported a study with a very similar experimental approach to ours. They also did patch-clamp recordings from infralimbic pyramidal neurons and analyzed mIPSCs, but they found *elevated* GABA release in their chronically stressed rats. They also reported *increased* numbers of inhibitory terminals onto pyramidal neurons together with decreased glucocorticoid receptor immunoreactivity specifically in PV+ interneurons (McKlveen et al., 2016). These findings are in sharp contrast to our present data showing impaired GABAergic neurotransmission. However, there are several dissimilarities between the experiment of McKlveen et al. (2016) and our present study which may explain the significant differences in the outcome. McKlveen et al. (2016) used different stressors, and their chronic variable stress paradigm lasted for only 14 days, which is much shorter compared to our present protocol, furthermore their stressors were also milder. Importantly, our earlier findings demonstrate that after two weeks of stress the HPA-axis is disturbed, but if stress is continued then corticosteron levels normalize again (Christiansen et al., 2012). Furthermore, McKlveen et al. (2016) recorded from layer V pyramidal neurons, whereas we studied layer II–III neurons. Our data suggest that longer lasting stress ultimately leads to impairments of the GABAergic network and points to cortical layer specific differences in the stress response.

We have been studying the effect of stress on GABAergic neurons for many years and we have repeatedly documented that stress can disrupt the functional and morphological integrity of GABAergic neurons in the hippocampus (Czéh et al., 2005, 2016; Hu et al., 2010, 2011; Holm et al., 2011; Nieto-Gonzalez et al., 2015; Csabai et al., 2017) and in the orbitofrontal cortex (Varga et al., 2017). It appears that the effect of stress on GABAergic neurons is region specific. In the hippocampus stress had a pronounced effect by reducing the number of PV+, CR+, NPY+ and SST+ neurons (Czéh et al., 2005, 2015; Hu et al., 2010; Csabai et al., 2017), while in the orbitofrontal cortex it was mainly the CB+ neurons which were affected by stress (Varga et al., 2017). Importantly, GABAergic neurons of the primary motor cortex were unaffected by stress (Varga et al., 2017).

In the hippocampus, we found ample evidences for impaired GABAergic neurotransmission (Holm et al., 2011; Hu et al., 2011; Nieto-Gonzalez et al., 2015) and impairment of GABAergic network functions (Hu et al., 2010). Others have also reported on stress-induced morphological and functional changes of GABAergic neurons in the hippocampus and neocortex (Gilabert-Juan et al., 2013, 2016; Maguire, 2014; MacKenzie and Maguire, 2015; Lee et al., 2016). Overall, it appears that stress affects GABAergic networks in multiple brain areas. We propose that these stress-induced deficits of inhibitory networks underlie the altered oscillation patterns that are implicated in cognitive impairments, which are common in patients with stress-related psychiatric illnesses.

In the present study, we used the object-place paired-associate learning task, to assess the effect of long-term stress on the cognitive capacities of the animals. This task was developed as a behavioral touchscreen task in which rodents learn to associate objects (shapes) with their specific locations on the screen (Talpos et al., 2009). In this PAL task the number of consecutive correct trials within one session is a combined readout on working memory, visual attention and executive functions. Anhedonic rats were significantly impaired in this task indicating disrupted functioning of hippocampal-prefrontal cortical circuits which are thought to underlie object-place learning (Warburton and Brown, 2010).

Our present study has several limitations. Touch-screen testing and* ex vivo* electrophysiological recordings are both very time-consuming methods. For logistic reasons it was not possible in every experiment to include both sub-groups of stressed rats (anhedonic and stress-resilient). The CMS protocol is labor demanding and only a fraction of the stress exposed rats displays a clear anhedonic or stress-resilient phenotype. During the data collection it became obvious that stress exposure has a significant effect mainly on anhedonic animals, therefore we focused on this subgroup.

Another limitation of this study is that the cognitive testing of the stressed rats was at a later time point compared to the electrophysiological measurements (see Figure 1A for the timeline of the experiment). Ideally, these experiments should have been done parallel to each other. However, due to logistic reasons we were not able to do that.

We focused here on the inhibitory network of interneurons in the neocortex, but the identification of GABAergic neurons can be a challenging task. Immunohistochemical studies using various antibodies against GABA or the rate-limiting enzyme, GAD, can yield different or even contradictory results (Houser, 2007). The most reliable method to visualize the entire population of GABAergic neurons is the *in situ* hybridization technique detecting either GAD65 or GAD67 mRNA (Houser et al., 2000; Czéh et al., 2013). In practice, neocortical GABAergic neurons are typically visualized by the use of various well-described cellular markers. In the present study we used six antibodies which collectively label almost the entire population of GABAergic cells. However, these cellular markers may occasionally label other cell types as well. There are reports on CB+ pyramidal cells which have been observed mainly in layer III of a variety of cortical areas (DeFelipe, 1997). In the present study, CB also labeled pyramidal neurons. This is clearly visible on Figure 7. During the cell counting, we counted only those CB+ neurons which had strong expression of CB and were multipolar (had dendritic processes projecting to all directions), in other words cells that had the morphological features of interneurons. However, we can not exclude the possibility that during our quantification of the CB+ neurons, a fraction of them were in fact pyramidal neurons.

In conclusion, our data demonstrate that long-term stress can induce depressive-like behavior in specific individuals and this behavioral phenotype is accompanied by pronounced structural and functional changes affecting the integrity of prefrontal GABAergic networks. Overall, this supports the concept that fronto-limbic GABAergic dysfunctions contribute to the behavioral and cognitive symptoms of MDD. Our data also suggest that prefrontal NPY+ neurons have a specific role in stress-coping abilities.

## Author Contributions

BC, IV, KJ and OW had the experimental concepts and designed the experiments. IV did all the electrophysiological recordings. ZV did all the cell counting. FF, KHøjgaard, KHenningsen and EB carried out the stress procedures and the behavioural testing. L-SM did the cognitive testing. DC did all the immunohistochemistry. AM contributed to the interpretation of the data. All authors contributed to the writing of the paper and revising it critically for important intellectual content. All author approved the final version to be published and agreed to be accountable for all aspects of the work in ensuring that questions related to the accuracy or integrity of any part of the work are appropriately investigated and resolved.

## Conflict of Interest Statement

The authors declare that the research was conducted in the absence of any commercial or financial relationships that could be construed as a potential conflict of interest.
