# Supplementary material for: Long-Term Stress Disrupts the Structural and Functional Integrity of GABAergic Neuronal Networks in the Medial Prefrontal Cortex of Rats
Source: Front Cell Neurosci. 2018 Jun 20;12:148. doi: 10.3389/fncel.2018.00148 (PMC6020798; doi:10.3389/fncel.2018.00148)
Supplement: Supplementary file 1 [file Data_Sheet_1.docx]

Supplementary Material

**Long-term stress disrupts the structural and functional integrity of GABAergic neuronal networks in the medial prefrontal cortex of rats.**

**Boldizsár Czéh *, Irina Vardya, Zsófia Varga, Fabia Febbraro, Dávid Csabai, Lena S. Martis, Kristoffer Højgaard, Kim Henningsen, Elena V. Bouzinova, Attila Miseta, Kimmo Jensen and Ove Wiborg**

*** Correspondence: Boldizsár Czéh, E-mail address: czeh.boldizsar@pte.hu**

# Supplementary Data

**Results of the correlation analysis for sucrose intake versus cognitive performance of the animals in the object-place paired-associate learning task.**

The graph shows the mean of two sucrose intake around the time point when the sessions of the maximum consecutive correct trials (CCM) were acquired. Sucrose intake correlated with the mean of maximum consecutive correct trials in row of session 28-30 OR the last three session an animal completed (8 out of 19 animals needed less than 30 sessions to complete the task).

We found a correlation of 0.53 and p = 0.020.


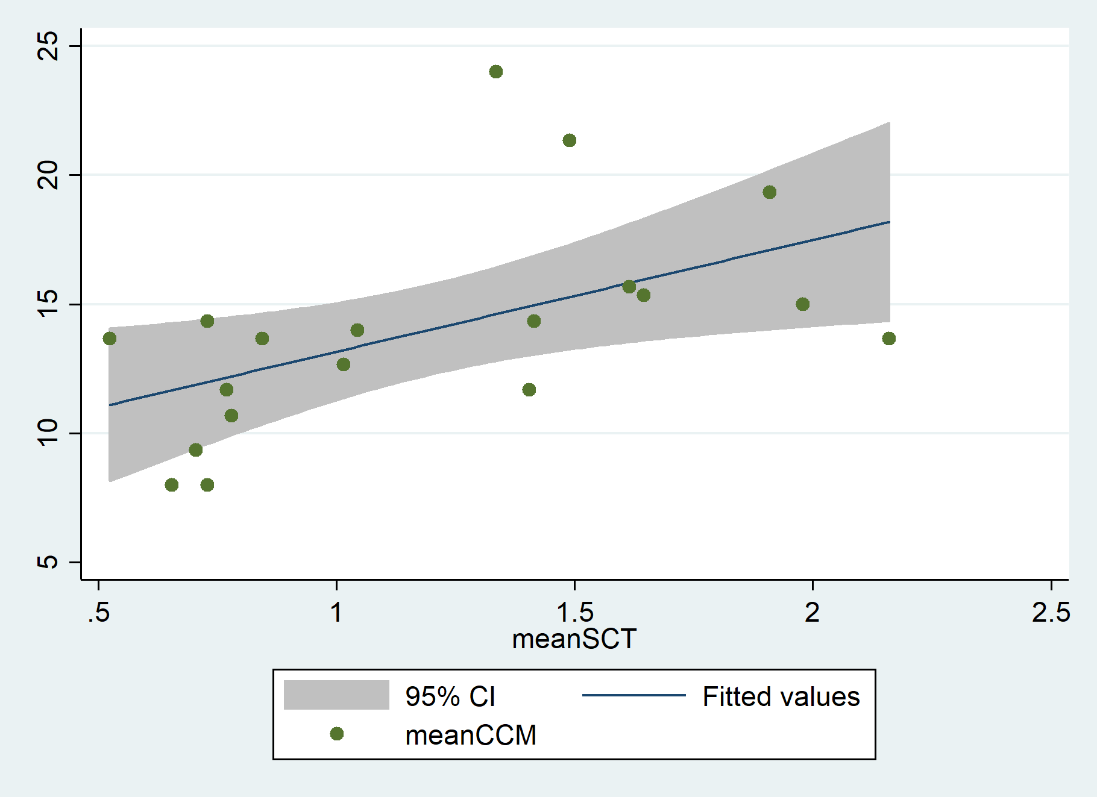


# Supplementary Tables

**Table 1.**

**Electrophysiological properties of layer II-III pyramidal neurons of the ventral mPFC**

| Parameter | **Control** | **Anhedonic** |
| --- | --- | --- |
| RMP | -74.8 ± 1.1 | -70.4 ± 1.3 |
| Capacitance (pF) | 25.5 ± 2.0 | 27.3 ± 2.5 |
| Input resistance (MΩ) | 83 ± 5 | 84 ± 5 |
| V_threshold_ (mV) | -31.9 ± 2.1 | -31.1 ± 1.7 |
| Reobase current (pA) | 355 ± 22 | 280 ± 21* |
| AP amplitude (mV) | 77.6 ± 2.7 | 77.4 ± 2.7 |
| AP Rise Time_10-90_ (µs) | 573 ± 15 | 543 ± 8 |
| AP duration at half-width (ms) | 1.5 ± 0.05 | 1.4 ± 0.05 |
| Delay of the first spike (ms) | 155 ± 51 | 95 ± 23 |

Summary of passive and active electrophysiological properties of layer II-III pyramidal neurons of the mPFC from control (*n_cells_* =10) and anhedonic rats (*n_cells_* = 8). The rheobase current was significantly lower in anhedonic-like rats, indicating that the pyramidal neurons display increased excitability. Values are mean ± SEM for all parameters. Statistics: unpaired two-tailed *t-*test, **P* < 0.05.

**Table 2.**

**The number of parvalbumin-positive neurons in the medial prefrontal cortex: Individual values**

|  | Control | Anhedonic | Stress-Resilient |
| --- | --- | --- | --- |
| anterior cingulate (Cg1) | rat_1_ = 8 839  rat_2_ = 8 088  rat_3_ = 6 626  rat_4_ = 7 424  rat_5_ = 7 093  rat_6_ = 7 538 | rat_1_ = 7 064  rat_2_ = 5 795  rat_3_ = 7 752  rat_4_ = 7 137  rat_5_ = 7 773  rat_6_ = 7 104 | rat_1_ = 7 129  rat_2_ = 8 017  rat_3_ = 5 590  rat_4_ = 7 540  rat_5_ = 7 568  rat_6_ = 8 102 |
| pre-limbic (PrL) | rat_1_ = 10 956  rat_2_ = 8 418  rat_3_ = 8 517  rat_4_ = 9 354  rat_5_ = 8 510  rat_6_ = 8 842 | rat_1_ = 8 149  rat_2_ = 6 368  rat_3_ = 9 313  rat_4_ = 7 877  rat_5_ = 8 782  rat_6_ = 8 098 | rat_1_ = 7 790  rat_2_ = 8 587  rat_3_ = 6 275  rat_4_ = 8 349  rat_5_ = 8 580  rat_6_ = 11 146 |
| infra-limbic (IL) | rat_1_ = 3 325  rat_2_ = 1 904  rat_3_ = 2 350  rat_4_ = 2 177  rat_5_ = 1 992  rat_6_ = 2 332 | rat_1_ = 1 543  rat_2_ = 992  rat_3_ = 1 776  rat_4_ = 1 246  rat_5_ = 1 608  rat_6_ = 1 433 | rat_1_ = 1 450  rat_2_ = 1 879  rat_3_ = 977  rat_4_ = 1 800  rat_5_ = 1 999  rat_6_ = 3 156 |

**Table 3.**

**The number of cholecystokinin-positive neurons in the medial prefrontal cortex: Individual values**

|  | Control | Anhedonic | Stress-Resilient |
| --- | --- | --- | --- |
| anterior cingulate (Cg1) | rat_1_ = 1 230  rat_2_ = 842  rat_3_ = 845  rat_4_ = 687  rat_5_ = 588  rat_6_ = 813 | rat_1_ = 571  rat_2_ = 690  rat_3_ = 590  rat_4_ = 518  rat_5_ = 586  rat_6_ = 688 | rat_1_ = 845  rat_2_ = 701  rat_3_ = 647  rat_4_ = 813  rat_5_ = 477  rat_6_ = 617 |
| pre-limbic (PrL) | rat_1_ = 1 582  rat_2_ = 1 168  rat_3_ = 1 077  rat_4_ = 1 018  rat_5_ = 722  rat_6_ = 1 098 | rat_1_ = 970  rat_2_ = 881  rat_3_ = 898  rat_4_ = 453  rat_5_ = 664  rat_6_ = 897 | rat_1_ = 926  rat_2_ = 1 038  rat_3_ = 889  rat_4_ = 1 176  rat_5_ = 654  rat_6_ = 804 |
| infra-limbic (IL) | rat_1_ = 666  rat_2_ = 465  rat_3_ = 348  rat_4_ = 235  rat_5_ = 123  rat_6_ = 345 | rat_1_ = 183  rat_2_ = 201  rat_3_ = 193  rat_4_ = 102  rat_5_ = 176  rat_6_ = 135 | rat_1_ = 158  rat_2_ = 269  rat_3_ = 175  rat_4_ = 279  rat_5_ = 127  rat_6_ = 194 |

**Table 4.**

**The number of calbindin-positive neurons in the medial prefrontal cortex: Individual values**

|  | Control | Anhedonic | Stress-Resilient |
| --- | --- | --- | --- |
| anterior cingulate (Cg1) | rat_1_ = 4 298  rat_2_ = 3 117  rat_3_ = 4 863  rat_4_ = 5 132  rat_5_ = 4 731  rat_6_ = 3 309 | rat_1_ = 3 299  rat_2_ = 4 271  rat_3_ = 3 135  rat_4_ = 2 610  rat_5_ = 2 505  rat_6_ = 4 013 | rat_1_ = 2 616  rat_2_ = 3 581  rat_3_ = 2 797  rat_4_ = 4 310  rat_5_ = 3 704  rat_6_ = 5 907 |
| pre-limbic (PrL) | rat_1_ = 8 467  rat_2_ = 7 904  rat_3_ = 7 459  rat_4_ = 7 358  rat_5_ = 7 539  rat_6_ = 6 931 | rat_1_ = 6 549  rat_2_ = 6 275  rat_3_ = 6 060  rat_4_ = 4 553  rat_5_ = 5 593  rat_6_ = 7 692 | rat_1_ = 4 507  rat_2_ = 5 625  rat_3_ = 4 420  rat_4_ = 7 452  rat_5_ = 7 153  rat_6_ = 10 199 |
| infra-limbic (IL) | rat_1_ = 3 219  rat_2_ = 3 266  rat_3_ = 2 471  rat_4_ = 2 645  rat_5_ = 3 677  rat_6_ = 2 080 | rat_1_ = 2 609  rat_2_ = 2 320  rat_3_ = 2 909  rat_4_ = 2 114  rat_5_ = 2 527  rat_6_ = 3 134 | rat_1_ = 2 352  rat_2_ = 2 365  rat_3_ = 2 450  rat_4_ = 2 961  rat_5_ = 2 982  rat_6_ = 3 544 |

**Table 5.**

**The number of calretinin-positive neurons in the medial prefrontal cortex: Individual values**

|  | Control | Anhedonic | Stress-Resilient |
| --- | --- | --- | --- |
| anterior cingulate (Cg1) | rat_1_ = 2 486  rat_2_ = 2 431  rat_3_ = 3 194  rat_4_ = 3 447  rat_5_ = 4 413  rat_6_ = 2 867 | rat_1_ = 2 802  rat_2_ = 2 804  rat_3_ = 2 098  rat_4_ = 2 106  rat_5_ = 2 452  rat_6_ = 2 396 | rat_1_ = 1 783  rat_2_ = 2 315  rat_3_ = 2 183  rat_4_ = 2 154  rat_5_ = 4 304  rat_6_ = 2 333 |
| pre-limbic (PrL) | rat_1_ = 4 117  rat_2_ = 4 083  rat_3_ = 4 842  rat_4_ = 3 883  rat_5_ = 4 303  rat_6_ = 4 245 | rat_1_ = 3 958  rat_2_ = 4 581  rat_3_ = 3 200  rat_4_ = 3 896  rat_5_ = 3 910  rat_6_ = 3 868 | rat_1_ = 3 591  rat_2_ = 3 581  rat_3_ = 3 327  rat_4_ = 3 852  rat_5_ = 5 679  rat_6_ = 4 080 |
| infra-limbic (IL) | rat_1_ = 1 481  rat_2_ = 1 275  rat_3_ = 1 501  rat_4_ = 1 836  rat_5_ = 1 701  rat_6_ = 1 558 | rat_1_ = 1 255  rat_2_ = 1 266  rat_3_ = 1 304  rat_4_ = 1 189  rat_5_ = 1 253  rat_6_ = 1 176 | rat_1_ = 1 114  rat_2_ = 1 177  rat_3_ = 1 195  rat_4_ = 978  rat_5_ = 1 435  rat_6_ = 1 180 |

**Table 6.**

**The number of neuropeptide-Y-positive neurons in the medial prefrontal cortex: Individual values**

|  | Control | Anhedonic | Stress-Resilient |
| --- | --- | --- | --- |
| anterior cingulate (Cg1) | rat_1_ = 2 326  rat_2_ = 1 777  rat_3_ = 2 121  rat_4_ = 1 747  rat_5_ = 1 744  rat_6_ = 1 943 | rat_1_ = 1 621  rat_2_ = 1 859  rat_3_ = 1 427  rat_4_ = 1 107  rat_5_ = 1 810  rat_6_ = 2 437 | rat_1_ = 3 310  rat_2_ = 2 118  rat_3_ = 1 980  rat_4_ = 2 149  rat_5_ = 2 361  rat_6_ = 2 845 |
| pre-limbic (PrL) | rat_1_ = 2 200  rat_2_ = 1 913  rat_3_ = 1 873  rat_4_ = 1 983  rat_5_ = 1 736  rat_6_ = 1 971 | rat_1_ = 1 868  rat_2_ = 2 205  rat_3_ = 1 718  rat_4_ = 1 407  rat_5_ = 1 946  rat_6_ = 2 660 | rat_1_ = 3 459  rat_2_ = 2 554  rat_3_ = 1 988  rat_4_ = 2 671  rat_5_ = 2 548  rat_6_ = 3 157 |
| infra-limbic (IL) | rat_1_ = 625  rat_2_ = 381  rat_3_ = 437  rat_4_ = 298  rat_5_ = 605  rat_6_ = 469 | rat_1_ = 365  rat_2_ = 518  rat_3_ = 454  rat_4_ = 514  rat_5_ = 462  rat_6_ = 537 | rat_1_ = 1 109  rat_2_ = 862  rat_3_ = 680  rat_4_ = 613  rat_5_ = 558  rat_6_ = 652 |

**Table 7.**

**The number of somatostatin-positive neurons in the medial prefrontal cortex: Individual values**

|  | Control | Anhedonic | Stress-Resilient |
| --- | --- | --- | --- |
| anterior cingulate (Cg1) | rat_1_ = 3 370  rat_2_ = 3 184  rat_3_ = 3 387  rat_4_ = 4 346  rat_5_ = 4 554  rat_6_ = 3 592 | rat_1_ = 3 428  rat_2_ = 2 849  rat_3_ = 3 852  rat_4_ = 3 481  rat_5_ = 4 004  rat_6_ = 4 029 | rat_1_ = 3 310  rat_2_ = 2 118  rat_3_ = 1 980  rat_4_ = 2 149  rat_5_ = 2 361  rat_6_ = 2 845 |
| pre-limbic (PrL) | rat_1_ = 5 587  rat_2_ = 5 429  rat_3_ = 4 713  rat_4_ = 6 226  rat_5_ = 6 046  rat_6_ = 5 202 | rat_1_ = 5 014  rat_2_ = 4 403  rat_3_ = 5 421  rat_4_ = 5 761  rat_5_ = 4 168  rat_6_ = 6 055 | rat_1_ = 3 459  rat_2_ = 2 554  rat_3_ = 1 988  rat_4_ = 2 671  rat_5_ = 2 548  rat_6_ = 3 157 |
| infra-limbic (IL) | rat_1_ = 1 664  rat_2_ = 1 538  rat_3_ = 1 307  rat_4_ = 1 663  rat_5_ = 1 968  rat_6_ = 1 581 | rat_1_ = 1 395  rat_2_ = 1 190  rat_3_ = 1 587  rat_4_ = 1 731  rat_5_ = 1 445  rat_6_ = 1 816 | rat_1_ = 1 508  rat_2_ = 2 110  rat_3_ = 1 592  rat_4_ = 1 919  rat_5_ = 1 799  rat_6_ = 1 975 |
